# Anticancer activities and mechanisms of heat-clearing and detoxicating traditional Chinese herbal medicine

**DOI:** 10.1186/s13020-017-0140-2

**Published:** 2017-07-12

**Authors:** Yulin Zhang, Yeer Liang, Chengwei He

**Affiliations:** State Key Laboratory of Quality Research in Chinese Medicine, Institute of Chinese Medical Sciences, University of Macau, N22-7038, Avenida da Universidade, Taipa, Macao, 999078 China

**Keywords:** Traditional Chinese medicine, Heat-clearing and detoxicating herbs, Anticancer, cellular and molecular mechanisms

## Abstract

In traditional Chinese medicine (TCM) theory, pathogenic heat and toxins, which are akin to the inflammatory factors, are the causes of cancer and could promote its virulent development. Therefore, heat-clearing and detoxicating (HCD) herbs are essential components of TCM formulas for cancer treatment. An increasing interest has been focused on the study of HCD herbs and accumulated evidences have shown that HCD herbs or HCD herbs-based formulas exhibited remarkable anticancer effects when used alone or combined with other therapeutic approaches. Some of the HCD herb-derived products have been tested in clinical trials. Studies revealed that extracts or pure compounds of the HCD herbs showed a broad anticancer spectrum against both solid and hematologic malignancies without significant toxic effects. Notably, some HCD herbs or formulas could strongly enhance the anticancer activities of chemo- or radio-therapy and alleviate their side effects. The anticancer activities of HCD herb exacts or the pure compounds were reported to be through multiple cellular or molecular mechanisms, such as induction of cancer cell apoptosis, differentiation and cell cycle arrest, inhibition of cancer cell growth, invasion and metastasis, and inhibition of tumor angiogenesis. In this review, we provide comprehensive analysis and summary of research progress and future prospects in this field to facilitate the further study and application of HCD herbs.

## Background

Cancers have been becoming one of the top killers worldwide. There were approximately 14.1 million new cancer cases and 8.2 million deaths from cancers in the world in 2012 according to the WHO statistics. Cancer still viciously scares people more than any other diseases despite substantial development of cancer diagnosis and treatment has been made. The majority of cancer patients are often diagnosed after the cancer has reached a terminal stage, at which chemotherapy is largely relied on. Although chemotherapy may temporarily slow tumor growth, they often lose the effectiveness as the cancer cells develop drug resistant. Some remedies may not be suitable for long-term use due to severe side effects. Thus, it is important to develop novel effective and safe approaches for cancer treatment. Comparing to modern Western medicine, traditional Chinese medicine (TCM) comprises a particularly safe and effective strategy in the treatment of cancer. In TCM theory, disequilibrium between Yin and Yang and blockage of meridian and viscera caused by interior (long time stress, anxiety, depress, overwork, improper lifestyle, etc.) and exterior factors (physical and chemical hazards) leads to stasis of Chi (vital energy), blood, dampness and phlegm, where the pathogenic heat and toxins, which are similar to the factors that cause prominent inflammation, are generated and promote occurrence and development of cancer eventually after these long-lasting malfunctions. Therefore, heat-clearing and detoxicating (HCD) herbs, Chi-regulating herbs, circulation-enhancing herbs, dampness and phlegm-resolving herbs are often used to treat cancers in TCM. HCD herbs are mostly cold in nature and bitter in taste and commonly used to clear away heat, purge fire, dry dampness and cool blood, and relieve toxins. Since pathogenic heat and toxins are more directly related to cancer, HCD herbs or formulas play a predominant role in cancer management by TCM. This review aims to summarize the representative anticancer HCD herbs and formulas, with emphasis on discussing the anticancer activities and the molecular mechanisms.

## Representative anticancer HCD herbs

The following representative anticancer HCD herbs are discussed in details: *Scutellariae* Radix (Huang Qin)*, Coptidis* Rhizome (Huang Lian)*, Artemisiae annuae* Herba (Qing Hao), *Hedyotis diffusa* (Bai Hua She She Cao)*, Rabdosiae rubescentis* Herba (Dong Ling Cao), and *Scutellariae barbatae* Herba (Ban Zhi Lian), which are very commonly prescribed HCD herbs in the anticancer TCM formulas and have been extensively studied.

### *Scutellariae* Radix

**Fig. 1:**
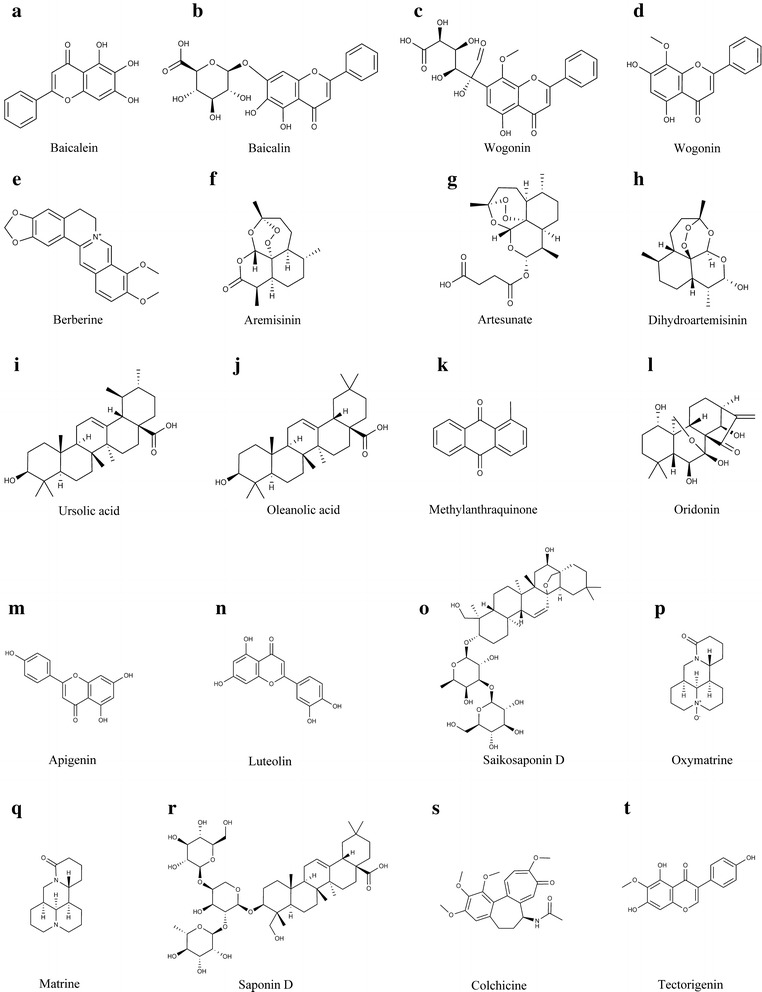
Chemical structures of major anticancer compounds in the representative HCD herbs

*Scutellariae* Radix (SR) is the dried root of *Scutellaria baicalensis* Georgi of the Lamiaceae family. SR is traditionally used to clear away pathogenic heat and activate blood circulation to remove stasis. Clinically, SR has long been used to treat pneumonia, jaundice, hypertension, dysentery and intestinal catarrh, pyogenic infection, etc. It is often prescribed in combination with other herbs in TCM formulas, such as Huang Qin Tang, Huang Qin Shao Yao Tang, and Huang Qin Mu Dan Tang. The most abundant compounds in SR are flavonoids, of which baicalein, baicalin, wogonoside and wogonin (Fig. 1a–d) showed strong anticancer activities.

Baicalein, the major flavone in SR, exhibited multiple pharmacological activities, such as anti-hepatotoxicity, anti-viral, anti-inflammation, and anticancer. Baicalein was reported to have anticancer activity against a wide spectrum of cancers [1], including esophagus, gastric, colorectal, pancreatic, lung, breast, ovarian, prostate and skin cancers [2, 3]. The anticancer activity of baicalein was through multiple mechanisms, e.g. suppressing hyperproliferation, inflammation, and metastasis, inducing apoptosis, etc. [3, 4], in which the PI3K/Akt and p38 pathways were engaged [5].

Wogonin, another flavone derived from SR, was proved to be effective in anticancer both in vitro and in mouse models, through inducing apoptosis, cell cycle arrest, and differentiation of cancer cells, inhibiting angiogenesis of tumor, and reversing drug resistance. Polier et al. reported that wogonin specifically inhibited the activity of cyclin-dependent kinase 9 (CDK9) and down-regulated the short-lived anti-apoptotic protein myeloid cell leukemia 1 (Mcl-1), which resulted in apoptosis in cancer cells [6]. Wogonin also induced nasopharyngeal carcinoma (NPC) cell apoptosis via inhibiting the activity of glycogen synthase kinase 3β (GSK-3β), a multifunctional serine/threonine kinase that was reported to inhibit apoptosis, and down-regulating the expression of ΔNp63, a survival factor in NPC cells [7]. In addition, wogonin inhibited tumor angiogenesis by promoting the degradation of hypoxia-inducible factors α (HIF-1α) via increasing its prolyl hydroxylation [8]. Acquired drug resistance is a serious problem in cancer treatment. Wogonin could reverse drug resistance in MCF-7/DOX cells through inhibiting the cell survival factors nuclear factor erythroid 2-related factor 2 (Nrf2) and heme oxygenase-1 (HO-1) [9]. Notably, wogonin significantly potentiated etoposide-induced apoptosis by impairing the function of P-glycoprotein and then increased cellular content of etoposide in HL-60 cells [10]. This synergistic effects were also observed when combination with fluorouracil in human gastric model. The synergistic anticancer activity of wogonin could be due to its pro-apoptotic effect and downregulation of NF-κB [11]. Furthermore, wogonin preferentially killed cancer cells instead of influence on normal cells. Based on these researches, *Scutellariae radix* and its effective constituents may serve as a clinically potential therapeutic agents against aggressive malignancies.

### *Coptidis* Rhizoma

*Coptidis* Rhizoma (CR) is the dried rhizome of *Coptis Chinese Franch.* of the Ranunculaceae family. The properties of CR are: extremely bitter and cold in nature, very strong in clearing heat and dampness, and detoxication. CR is commonly used in China for the treatment of gastroenteritis, liver disease, hypertension, and other inflammatory diseases accompanied by high fever. CR or its components were found to be beneficial for a wide range of diseases, such as diarrhea, pressure-overload induced cardiac hypertrophy, hypercholesterolemia, atherosclerosis, Alzheimer’s disease, and diabetes mellitus. Interestingly, our studies and others demonstrated that CR extract exhibited strong anticancer effects in vitro and in vivo used alone or combined with chemotherapeutic drugs [12–14]. CR extract significantly inhibited tumor growth and colony formation of gastric, colon, and breast cancer cells. Breast cancer cells were particularly sensitive to CR. The growth inhibition was associated with suppression of cyclin B1 protein, which resulted in complete inhibition of CDC2 kinase activity and cell cycle arrest at G_2_ phase [15]. Iizuka and his colleagues reported that the aqueous extract of CR exhibited inhibitory effect on the proliferation of esophageal cancer cells and arrested the cells at G_0_/G_1_ phase [16]. CR supplementation significantly attenuated weight loss in tumor-bearing nude mice without changing food intake or tumor growth, and maintained good nutritional status in these mice. The anti-cachectic effect was accompanied by significantly reduced interleukin 6 (IL-6) expression [17].

The most abundant compounds in CR are alkaloids, of which berberine (Fig. 1e) is the most important active compound, with its dry weight consisting of up to 7.1 mg/100 mg of CR [18]. Recent data have shown that berberine was applied in treating inflammation, cancer, colitis, diabetes, high blood cholesterol etc. [19]. Considerable attention has been drawn to berberine since its prominent anticancer activity including tongue squamous cancer, esophageal cancer, hepatocelluar cancer, breast cancer, lung cancer, gastric cancer, ovarian cancer, renal cancer, nasopharyngeal cancer and Wilms’ tumor [20]. Berberine has been proved to be a heat-clearing and detoxifying compound which acts on diverse cancer cell types through various mechanisms. For the treatment of colorectal cancer, berberine was mainly involved in inducing apoptosis and restraining inflammation, inhibiting tumor growth, inactivating Wnt/β-catenin signaling, promoting the generation of ROS, inhibiting arylamine N-acetyltransferase (NAT) activity and cyclooxygenase 2 (COX-2) expression [21]. Interestingly, berberine significantly reduced the familial adenomatous polyposis patients’ polyp size through inhibition of Wnt signaling, suggesting an application in the prevention of colon cancer [22]. Berberine also suppresses the invasive and metastasis of nasopharyngeal carcinoma (NPC) by inhibiting the activation of Signal Transducer and Activator of Transcription 3 (STAT3), a key mediator to trigger tumor-promoting inflammation [23]. The similar actions were observed in lung cancer, of which cell proliferation and tumor spheroid formation were suppressed by berberine [24]. Notably, berberine exhibits selective cytotoxicity against cancer cells instead of normal hepatocytes [25]. In human breast cancer cells, berberine induces apoptosis through a mitochondrial dependent pathway by increasing the Bax/Bcl-2 protein ratio, activating caspases and inducing poly (ADP-ribose) polymerase (PARP) cleavage [26]. The induction of interferon β and tumor necrosis factor (TNF) α in cancer cells is responsible for the anti-breast cancer activity of berberine [13]. Furthermore, berberine significantly enhanced the anticancer effect of estrogen receptor (ER) antagonists on ER positive breast cancer cells through down-regulating the expression of cancer related genes, such as epidermal growth factor receptor (EGFR), human epidermal growth factor receptor 2 (HER2), and bcl-2 [14]. Improvement of the chemo- and radio-sensitivity of tumors by herbs indicates another strategy of treatment in cancer therapy. Anticancer efficacy was significantly enhanced when combining berberine with granted chemotherapeutic agents such as vincristine or 2-deoxy-d-glucose in certain cancer cells [27]. Combined with γ radiation, berberine exhibited pro-apoptotic effect in hepatocellular carcinoma cells [28]. The chemosensitization of berberine was showed in colon cancer cells while the radiosensitization was obtained in esophageal squamous carcinoma cells, human nasopharyngeal carcinoma cells, and breast carcinoma cells [29, 30]. It’s important to note that berberine has so poor bioavailability that it can hardly be an independent anti-tumor agent [31]. Nevertheless, berberine could be a promising adjuvant to chemotherapy and radiotherapy of a wide range of cancers.

### *Artemisiae annuae* Herba

*Artemisiae annuae* Herba (AAH) is the dried aerial part of *Artemisia annua* L. of the Compositae family. It was initially used for treating fevers in TCM, and was then renowned to be an antimalarial herb. Recent studies indicated that AAH showed high potential anticancer activities [32].

The most abundant compounds in AAH are sesquiterpene lactones, of which artemisinin (Fig. 1f) is the most active compound. Artemisinin has a broad range of biological activities, such as anti-viral, anti-fungal, anti-parasitic, anti-inflammation, and anticancer. The anticancer activities of artemisinin include anti-proliferation, anti-angiogenesis, anti-invasion, anti-metastasis and cytotoxicity [33]. Artemisinin and its derivatives, such as artesunate (Fig. 1g) and dihydroartemisinin (DHA) (Fig. 1h), exhibit potential anticancer effects on various types of cancer cells, including breast cancer, leukemia, ovarian cancer, hematoma, prostate cancer, colon cancer, gastric cancer, melanoma and lung cancer [34]. Artemisinin was reported to inhibit angiogenesis through down-regulating the expression of vascular endothelial growth factor (VEGF), a key angiogenesis stimulator, in in vitro and in vivo assays [35]. Artemisinin induced a strong stringent G1 cell cycle arrest in prostate cancer cells, human breast cancer cells and nasopharyngeal cancer cells by down-regulating the expression of CDK2, CDK4, cyclin E, cyclin D1 and E2F1, and increasing the expression of p16 (also known as cyclin-dependent kinase inhibitor 2A) [36]. DHA treatment caused cervical cancer cell growth inhibition via upregulation of Raf kinase inhibitor protein (RKIP) and downregulation of bcl-2 [37]. Artemisinin can alter apoptosis-related protein expression which may further inhibit cell proliferation and induce apoptosis. Artemisinin downregulated IGF-IR expression and inhibited the growth of MCF-7 breast tumor cell xenografts in nude mice [38]. Moreover, the inhibition of Bcl-2 family, activation of Bax and release of cytochrome c in human colon cancer cells illuminate the pro-apoptotic mechanisms of artemisinin [39]. Artemisinin may also be a potential anti-metastasis agent against melanoma cells and hepatocarcinoma cells by reducing MMP2 level [40]. A more recent report revealed that DHA activates the autophagy program by suppressing the nuclear translocation of NF-κB [41]. In vivo experiments showed that oral administration of artemisinin at 50 mg/kg/day decreased tumor growth [40]. The toxicity of artemisinin remains a challenge for its development in the clinical application. In addition to possessing cytotoxicity in various tumors, artemisinin shows slight neurotoxicity and may cause drug resistance in vivo [33]. Besides, artemisinin and its derivatives produce synergistic anticancer effects in combination with other chemotherapeutic drugs. For instance, DHA sensitized human ovarian cancer cells to carboplatin therapy and synergistically enhanced the anticancer effect of gemcitabine on human lung cancer cells [42, 43].

### *Hedyotis diffusa*

*Hedyotis diffusa* (HD) is the dried whole plant of genus Saxifraga of the *Rubiaceae* family. As a well-known traditional Chinese folk medicine, it frequently appears in Chinese medicinal formulas and has long been used for heat-clearing, detoxification, promotion of blood circulation and removal of blood stasis [44]. Accumulating evidences indicate that HD possesses anticancer, antioxidative, hepatoprotective, neuroprotective, anti-inflammatory, anti-mutagenesis and immunoregulatory activities [45]. It is applied in the treatment of inflammation-related diseases, such as appendicitis, bronchitis and urethritis. Pharmacological studies propose that HD performs vital roles in the treatment of solid tumors, including liver, lung, colon, and other cancers [46]. Both organic and aqueous extracts of HD exhibit remarkable anticancer activities. The methanol extracts of HD can suppress cancer cell proliferation and induce apoptosis, which involve many tumor-related genes and proteins (e.g. TNF-α, IL-1, NF-κB, Fas, AP-1, Bcl-2, Bcl-xL) [45]. The ethanol extracts inhibit angiogenesis and induce mitochondrion-dependent apoptosis through PI3K/Akt and XIAP pathways [47]. The aqueous extracts inhibited HepG2 cell growth and enhanced the anticancer activity of 5-fluorouracil via suppressing CDK2-E2F1 activity [48].

Phytochemistry studies show that it contains components with anticancer activities, including anthraquinones, flavones, hemiterpenes, polyphenols, organic acids and polysaccharides [49], of which ursolic acid (Fig. 1i) and oleanolic acid (Fig. 1j) are two major anticancer compounds [46]. Ursolic acid demonstrated effective in anti-leukemia, which involves diverse biological functions, such as inhibition of cell growth, induction of cell differentiation and apoptosis [50]. The associated mechanisms include inactivation of protein kinase B (PKB), activation of c-Jun N-terminal kinases (JNK) and extracellular signal-regulated kinase (ERK) pathways, intracellular Ca^2+^ release, etc. Ursolic acid also exhibits therapeutic potential in the treatment of hormone refractory and androgen-sensitive prostate cancer through induction of cancer cell apoptosis via activation of JNK-induced Bcl-2 phosphorylation and degradation [51]. Methylanthraquinone (Fig. 1k), another active compound from HD, shows multiple anticancer effects on many cancer types. It induced apoptosis in human breast cancer MCF-7 cells by increasing intracellular calcium levels, activating JNK, calpain, and eventually caspases 4, 9, 7 [52]. Methylanthraquinone also caused apoptosis in human leukemic U937 cells by decreasing phospho-ERK1/2 and increasing phospho-p38 MAPKs [53]. Taking together, accumulating evidences indicates the therapeutic potential of HD or its components in treating various cancers.

### *Rabdosiae rubescentis* Herba

*Rabdosiae rubescentis* Herba (RRH) is the dried aerial part of genus *Rabdosia rubescens* (Hemsl.) Hara of the Lamiaceae family. RRH, or Dong Ling Cao in Chinese, which means “ice grass” due to its strong heat-clearing and detoxifying properties, is a well-known HCD herb possessing several biological activities, such as anti-bacteria, anti-parasites, anti-inflammation, and anticancer [54].

The chemical components of RRH are relatively complex, mainly including monoterpenes, sesquiterpene, diterpene and tripenoids. Oridonin (Fig. 1l), a tetracyclic terpenoid compound, is the main active component purified from RRH [55]. In recent years, increasing attention has been gained on oridonin due to its remarkable growth inhibition and apoptosis induction activities in cancer cells. In vitro and in vivo studies showed that oridonin induced apoptosis in cells derived from a variety of cancers, including hepatocellular carcinoma, breast cancer, skin cancer, colorectal cancer, gallbladder cancer, gastric cancer, pancreatic cancer and osteoma [56]. Notably, oridonin has less cytotoxicity to normal cells such as fibroblasts and lymphoid cells [56]. Oridonin could arrest cell cycle at the G2/M phase in hepatocarcinoma HepG2 cells by upregulating serine-threonine kinase receptor-associated protein, heat shock 70 kDa protein 1, stress-induced phosphoprotein 1, etc. [57]. Oridonin also drastically suppresses tumor invasion and metastasis in vitro via regulating the integrin β1/FAK pathway and decreasing the expression of MMPs in MDA-MB-231 cells in vitro [58]. A study on cervical cancer found that oridonin induced the apoptosis of cancer cells through PI3K/Akt pathway [59]. In another study on gastric cancer indicated that the mechanism of oridonin-induced apoptosis involved Apaf-1, cytochrome c and caspase-3 signaling pathway [60]. Accumulating studies have shown an enhanced anticancer effect when oridonin was combined with imatinib in Ph^+^ acute lymphoblastic leukemia cells. The results showed that oridonin inhibited the activations of LYN (one of SRC family kinases) and ABL and their downstream Raf/MEK/ERK, Akt/mTOR, and STAT5 pathways, decreased Bcl-2/Bax ratio and then induced apoptosis in Ph^+^ ALL cells [61]. In addition, some recent studies suggested that oridonin could also inhibit the proliferation of tumor cells by increasing the autophagy of tumor cells [62]. Current research on pancreatic cancer cells indicated that oridonin could induce apoptosis via p53- and caspase-dependent induction of p38 MAPK [63]. Meanwhile, apoptosis, autophagy and loss of the mitochondrial transmembrane potential have been observed in lung cancer cell line A549 treated with oridonin [64]. Therefore, oridonin is supposed to be a promising compound for chemotherapy.

### *Scutellariae barbatae* Herba

*Scutellariae barbatae* Herba (SBH) is the dried whole plant of genus *Scutellaria barbata* D. Don of the Lamiaceae family. SBH contains several flavonoids, alkaloids, polysaccharides, and steroids [65]. The extracts of SBH exhibited significant anticancer activities in several human cancers such as colon cancer, leukemia, hepatoma, skin cancer, breast cancer and chorioepithelioma [66]. Despite the distinguished success of this herb in treating cancer, the precise molecular mechanisms still remain to be investigated. Studies revealed that ethanol extract of SBH (ESBH) could induce apoptosis, inhibit proliferation and angiogenesis in colon cancer [67]. Administration of ESBH remarkably increased the levels of pro-apoptotic Bax/Bcl-2 ratio and the expression of suppressor gene p21, whereas decreased the expression of pro-proliferative genes Cyclin D1 and CDK4 [67]. Further studies on benign smooth muscle cell tumor model demonstrated that SBH could induce differentiation and apoptosis in uterine smooth muscle cells [68]. In addition, SBH showed well-validated chemopreventive activity at stages of initiation, promotion, and progression of cancer [69]. Increasing evidences have also revealed that the combination therapy of SBH with other commonly prescribed chemotherapeutic agents could considerably inhibit the growth of carcinoma both in vitro and in vivo [70]. Although the active anticancer constituents have not been identified, flavonoids in SBH have become the focus of researches, since this kind of compounds strongly inhibited cancer cell proliferation, induced mitochondria-dependent apoptosis, and inhibited tumor angiogenesis [65]. Nevertheless, more efforts are required to investigate the active compounds in SBH to facilitate the research and development of this promising anticancer herb.

### Summary of the major active compounds and their actions

**Fig. 2:**
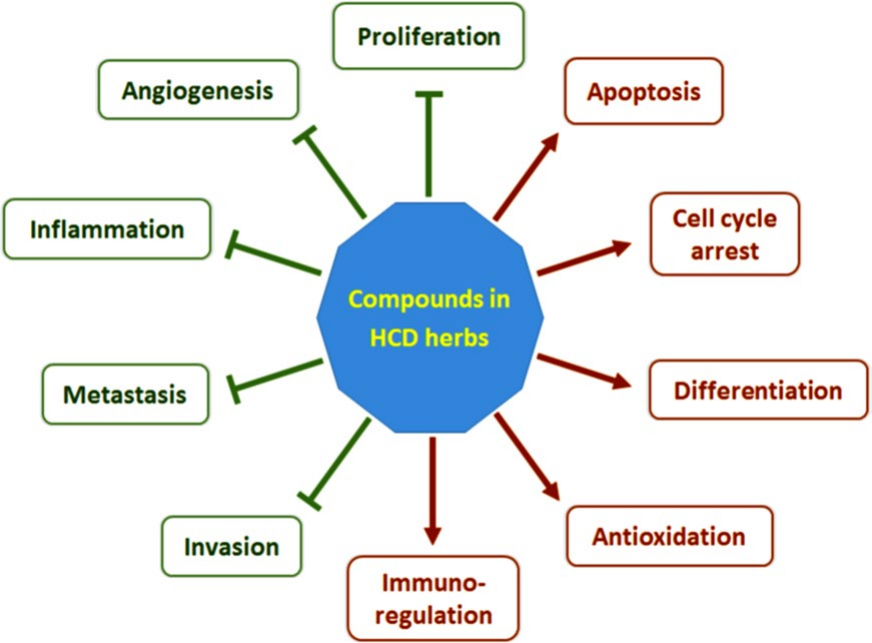
Schematic diagram showing the anticancer activities and mechanisms of compounds in HCD herbs

The chemical structures of major anticancer compounds in the representative HCD herbs are shown in Fig. 1. The anticancer activities and their mechanisms of the major compounds in the above-discussed and other typical HCD herbs are summarized in Table 1. The anticancer compounds in HCD herbs are comprised of various types of chemicals, including, but not limiting to, alkaloids (e.g. berberine, matrine and colchicine), flavonoids (e.g. baicalein, wogonin, luteolin, apigenin and tectorigenin), terpenoids (e.g. oridonin, ursolic acid, oleanolic acid, artemisinin and saikogenin), anthraquinones, polyphenols, organic acids (e.g. ursolic acid and oleanolic acid), polysaccharides, saponins (e.g. saikosaponin and pulsatilla saponins), etc. Studies indicated that the active compounds from HCD herbs exhibited multifarious anticancer activities, such as inhibition of proliferation, invasion, metastasis, inflammation, and angiogenesis, induction of differentiation, apoptosis and cell cycle arrest, antioxidation, and modulation of immune function (Fig. 2). The versatile anticancer effects of these compounds are also indicated by the potency against a broad spectrum of cancer types, both various solid tumors and hematopoietic malignancies. HCD herbs are primarily characterized by the heat-clearing and detoxicating properties in TCM theory, which correlate with their antioxidant activity. Indeed, most, if not all, compounds from HCD herbs are antioxidants, such as berberine, matrine, baicalein, polyphenols and polysaccharides. Since tumors frequently exhibit high levels of oxidative stress [71], a general disturbance of redox balance in cancer cells by HCD herbal compounds may contribute to their multifarious anticancer effects. In addition, these compounds could regulate a wide range of signaling pathways, kinase activity and gene expression, which are involved in cell proliferation, cell cycle, apoptosis, invasion, metastasis, etc. However, the anticancer potential and detailed molecular mechanisms of the compounds remain to be further elucidated.

**Table 1:** The major active components, anticancer effects and mechanisms of HCD herbs

HCD herbs	Major anticancer compounds	Cancer types (cell lines)	Cellular effects	Molecular mechanisms	References
*Scutellariae* Radix	Baicalein, wogonin	Esophageal squamous cell carcinoma (EC-109), glioma (U87MG, U251MG, C6, U251), colon cancer (HCT116), bladder cancer (TSGH8301, BFTC905, RT4, T24, HT1376), breast cancer (T47D, MCF-7, SK-BR-3, MDA-MB-231, SKBR3), leukemia (CEM), pancreatic carcinoma (Colo-357), hepatocellular carcinoma (HepG2, SK-HEP-1), Hodgkin’s lymphoma (L1236), melanoma (SK-MEL-37), nasopharyngeal carcinoma (NPC-TW076, NPC-TW039)	Inhibiting proliferation, invasion, migration and angiogenesis, inducing apoptosis and differentiation	↓MMP2, ↓MMP9, ↑TIMP1, ↑TIMP2, ↓p38, ↑PI3K/AKT, ↓NF-κB, ↑PPAP γ, ↓CDC2-survivin, ↓Wnt, ↓CDK9, ↓HIF-1α, ↓VEGF, ↑GSK3β/β-catenin, ↓ΔNp63, ↑cleaved PARP, ↑caspase-3, ↑caspase-7, ↑p21	[3–11, 22, 29]
*Coptidis* Rhizoma	Berberine	Breast cancer (MCF7, MDA-MB-468), gastric cancer (MKN-74), colon cancer (HCT116, SW480, SW620, DLD-1, KM12, KM12SM, KM12L4A,), esophageal cancer (YES-1, YES-2, YES-3, YES-4, YES-5, YES-6), nasopharyngeal carcinoma (5-8F, C666-1), kidney cancer (G401), bladder cancer (T24), hepatocellular carcinoma (HepG2)	Inhibiting proliferation, invasion, migration and angiogenesis, inducing apoptosis, cell cycle arrest at G0/G1 phase and mitochondrial membrane damage, increasing ROS	↑IFN-β, ↑TNF-α, ↓Cyclin B1, ↓CDC2, ↓Ezrin, ↑p27, ↑p21, ↑Cyclin E, ↑AMPK, ↑WTX, ↓GSK3β/β-catenin, ↓JNK/p38, ↓Wnt, ↓STAT3, ↑caspase-8, ↑caspase-3, ↓Bid, ↑Bax/Bcl-2, ↑Fas, ↑cleaved PARP, ↓NAT, ↓COX-2, ↑TRAIL, ↑VEGF	[13–16, 19–31]
*Artemisiae annuae* Herba	Artemisinin	Leukemia (HL-60, NB4), ovarian cancer (HO-8910), prostate cancer (LNCaP), breast cancer (MCF-7), nasopharyngeal carcinoma (CNE-1, CNE-2), hepatocellular carcinoma (HepG2, SMMC-7721), melanoma (A375P, A375M), myeloma (RPMI 8226), colon cancer (HCT16), cervical cancer (HeLa, Caski)	Inhibiting proliferation, invasion, migration and angiogenesis, Inducing apoptosis, cell cycle arrest at G1 phase and mitochondrial dysfunction, increasing ROS	↑MAPKs, ↓p38MAPK, ↓VEGF, ↓KDR, ↓CDK2, ↓CDK4, ↓cyclin E, ↓cyclin D1, ↓E2F1, ↓BMI-1, ↑RKIP, ↓Bcl-2, ↑Bax, ↑caspase-3, ↓IGF-IR, ↓MMP2, ↓TIMP2, ↓α_V_β_3_, ↓NF-κB	[34–43]
*Hedyotis diffusa*	Ursolic acid, methylanthraquinone	Histocytic lymphoma (U937), leukemia (HL-60), colon cancer (HT-29), melanoma (B16-F10), lung cancer (A549), breast cancer (MCF-7), prostate cancer (LNCaP), Tsu-Pr1, MDA-MB-453, DU-145, cervical cancer (C-33A, U14), sarcoma (S180), hepatocellular carcinoma (HepG2, H22, SMMC-7721)	Inhibiting proliferation, migration and angiogenesis, inducing differentiation, apoptosis, cell cycle arrest at G0/G1, S or G2/M phases, DNA fragmentation and mitochondrial dysfunction	↑interferon-γ, ↑TNF-α, ↑IL-1, ↓NF-κB, ↑Fas, ↑p53, ↑p21/Cip1, ↓p27/kip1, ↑caspase-3, ↑caspase-9, ↑AP-1, ↓Bcl-2, ↑Bax, ↓STAT3, ↓cyclin D, ↓cyclin D1, ↓cyclin D2, ↓cyclin E, ↓CDK4, ↓CDK2, ↓E2F1, ↑Fas-L, ↑TRAIL, ↓MAPK/ERK, ↓JNK, ↑PI3K, ↑p21/WAF1, ↑CDKN1A, ↓cyclin A2, ↓cyclin B1, ↓ODC1, ↓VEGF-α, ↑HSP 70, ↑P16, ↓pim-1, ↓rel, ↓ras, ↓fos, ↓myc, ↑IFN-γ	[45–51, 53]
*Rabdosiae rubescentis* Herba	Oridonin	Hepatocellular carcinoma (HepG2), gallbladder carcinoma (SGC996, NOZ), breast cancer (MCF-7, MDA-MB-231), cervical cancer (HeLa), histiocytic lymphoma (U937), pancreatic cancer (SW1990)	Inhibiting proliferation, migration and invasion, inducing apoptosis, autophagy, DNA damage and cell cycle arrest at S and G2/M phases, increasing ROS	↑Hsp70-1, ↑STAP, ↑TCTP, ↑Sti1, ↑PPase, ↓hnRNP-1, ↑HP1 β, ↑GlyRS, ↓NF-κB, ↑Bax/Bcl-2, ↑caspase-3, ↑caspase-9, ↑cleaved PARP, ↓IKKα, ↓IKKβ, ↓mTOR, ↑Fas, ↑PPAR-γ, ↓MMP2/MMP9, ↓Integrin β, ↓FAK, ↓Akt, ↓FOXO, ↓GSK3, ↓ERK, ↓IL-1β, ↑Beclin-1, ↑LC3 II/I, ↑Atg4B, ↓p38 MAPK, ↑p53, ↑p21	[53, 56, 57, 59–64]
*Scutellariae barbatae* Herba	Apigenin, luteolin	Hepatocellular carcinoma (MHCC97H), colon cancer (HT-29), leukemia (LM-1, LM-2), cervical cancer (HeLa)	Inhibiting proliferation and invasion, inducing apoptosis, differentiation and cell cycle arrest at G1 phase	↓MMP-2, ↓MMP-9, ↑TIMP-1, ↑TIMP-2, ↑ACTA2, ↑Calponin, ↑p27, ↓Cyclin D1, ↓CDK4, ↑p21, ↑Bax/Bcl-2, ↓STAT3, ↓ERK1/2, ↓p38, ↑Smac, ↑Apaf-1, ↑caspase-9, ↑caspase-3, ↓IGF-1, ↑cytochrome c	[65–70]
*Bupleuri* Radix	Saikosaponin D	Cervical cancer (HeLa), breast cancer (MCF-7), prostate cancer (PC3), lung cancer (H1299, LLC-1, A549), hepatocellular carcinoma (HepG2, Hep3B), gastric adenocarcinoma (MK-1), cervical cancer (HeLa), melanoma (B16F10), leukemia (P-388), oral epidermoid carcinoma (KB)	Inhibiting proliferation, invasion, metastasis, and angiogenesis, inducing apoptosis, autophagic cell death, cell cycle arrest at G1, or G2/M phases	↑ERK1/2, ↑caspase-3, ↑caspase-9, ↑caspase-7, ↑cleaved PARP, ↑AMPK, ↑mTOR, ↑p27, ↓p53, p21/WAF1, ↑Fas/APO-1, ↑mFasL, ↑sFasL, ↑Bax, ↓IKB-α, ↓NF-κB, ↓Bcl-XL, ↓SERCA Ca^2+^ pump, ↑[Ca^2+^]_i_, ↓telomerase, ↑tubulin polymerization	[91–93]
*Sophorae flavescentis* Radix	Matrine	Hepatocellular carcinoma (H22, S180, SMMC-7721, HepG2, Hep-7402), breast cancer (MA737, MKN45, SGC-70901, MDA-MB-231), gastric cancer (SGC-7901), melanoma (A375, SK-MEL-2, M21, B16-F10), cervical cancer (HeLa), leukemia (K-562), glioma (C6), lung cancer (A549, NCI-H460), ovarian cancer (SK-OV-3), central nervous system cancer (XF498), pulmonary adenoma (SPC-A-1), esophagus cancer (Eca-109), colon cancer (SW1116, HCT-15), osteosarcoma (UMR-108, MNNG/HOS), pancreatic cancer (PANC-1), leukmia (U937, HL-60), adenoid cystic carcinoma (ACC-M), retinoblastoma (Y79, WERI-RB1, SO-RB50), nasopharynx cancer (TW03)	Inhibiting proliferation, adhesion, invasion, metastasis and angiogenesis, inducing apoptosis, autophagy, differentiation and cell cycle arrest at G0/G1 or G2 phases, modulating immune function	↑Beclin 1, ↑Bax, ↓Bcl-2, ↑caspase-8, ↑caspase-3, ↑caspase-9, ↑AKT, ↓NF-κB, ↓IκBα, ↓p65, ↓ERK1/2, ↓JNK, ↓p38 MAPK, ↓TNFαl, ↓IKB-α, ↓p65, ↓ERK1/2, ↑E2F1, ↓Rb, ↑Apaf-1, ↓MMP-9, ↓MMP-2, ↓AKT, ↓EGF, ↓VEGF, ↓VEGF2, ↓VEGFR1, ↑Fas, ↑FasL, ↑p21, ↑p27, ↓Cyclin D1, ↓Cyclin E, ↓hTERT	[94, 95]
*Pulsatillae* Radix	Pulsatilla saponin A, D, H	Gastric cancer (MKN-45, MKN-28, AGS), colon cancer (HT-29, LoVo), hepatocellular carcinoma (Huh-7, HepG2)	Inducing DNA damage and apoptosis	↑Caspase-3, ↑cleaved PARP, ↑Bax, ↓c-Met, ↓AKT, ↓mTOR, ↓p70S6K, ↓HIF-1α, ↓VEGF	[96, 97]
*Cremastrae pseudobulbus, Pleiones pseudobulbus*	Colchicine	Hepatocellular carcinoma (HCC24/KMUH, HCC38/KMUH)	Inhibit proliferation interacting with tubulin	↑AKAP12, ↑TGF-β2, ↑MX1	[98, 99]
*Belamcandae* Rhizoma	Tectorigenin	Hepatocellular carcinoma (HepG2), lung cancer (LLC), sarcoma (S180), prostate cancer (LNCaP)	Inhibiting the proliferation and angiogenesis, inducing apoptosis, differentiation and mitochondrial dysfunction, increasing ROS	↑Caspase-3, ↑caspase-9, ↓PDEF, ↓PSA, ↓IGF-1, ↑TIMP-3, ↑cytochrome c, ↑[Ca^2+^]_i_	[100, 101]

## The representative anticancer formulas containing HCD herbs

Cocktails of medicines are usually applied to treat complex syndromes or diseases, like cancers and cardiovascular diseases. Similarly, single herb is seldom used in TCM, of which formulas with combination of various herbs are much more often prescribed on the basis of individual conditions according to TCM theory. Cancers often exhibit excessive heat and toxin. Therefore, one or more HCD herbs are the major components in anticancer TCM formulas. In recent years, the application of TCM prescriptions in the treatment of various malignant tumors has obtained encouraging outcomes, at least partially owing to that multiple components can act on multiple targets and exert synergistic therapeutic efficacies. In particular, apart from traditional decoction and oral administration methods, advanced pharmaceutical technologies are used in TCM formula preparations with distinctive advantages and features, including tablets, pills, capsules, injections, powder, liquids, etc. [72]. The following representative anticancer formulas containing HCD herbs are discussed in details, including Yanshu Injection, Huanglian Jiedu Tang, Jiedu Xiaozheng Yin and PHY906. Some others are listed in Table 2.

### Yanshu Injection

Yanshu Injection (YSI), also named Fu Fang Ku Shen injection, consists of two herbs: *Sophorae flavescentis* Radix (SFR, or Ku Shen) and *Smilacis glabrae* Rhizoma (SGR, or Tu Fu Ling) with the ratio of 7 to 3. Both of them belong to heat-clearing and detoxifying herbs. SFR is commonly used for the treatment of viral hepatitis, cancer, enteritis, viral myocarditis, arrhythmia, skin diseases, etc. The major anticancer compounds in SFR are oxymatrine (Fig. 1p) and matrine (Fig. 1q), which have been approved for the treatment of cancers by the Chinese State Food and Drug Administration (SFDA) [73]. The compounds showed broad spectrum of anticancer activities including stomach, esophagus, liver, colon, lung, cervix, ovary, and breast cancers, through multiple mechanisms, such as inhibiting cancer cell proliferation, inducing apoptosis and autophagy, modulating immune response, reducing cancer cell adhesion, invasion and migration [73]. SGR is widely used both in food supplementary and health care, owing to its properties of heat-clearing and detoxication. Studies reported its therapeutic potential for the treatment of rheumatoid arthritis, inflammation, liver injury, hyperinsulinemia and cancer [74]. Crude extraction of SGR as well as its pure compounds including astilbin, 5-*O*-caffeoylshikimic acid and taxifolin, could promote cancer cell apoptosis and block cancer cell adhesion, invasion and migration by inhibiting transforming growth factor beta 1 (TGF-β1) signaling pathway. YSI was reported to be able to directly inhibit gastric cancer cell proliferation and block the experimental gastric carcinogenesis by preventing carcinogen-induced oxidative damage and improving immune function [75]. However, YSI was mostly applied in combination with chemotherapy or radiotherapy in cancer treatment. Studies showed that YSI plus transcatheter arterial chemoembolization (TACE) could synergistically enhance the therapeutic effects of TACE, alleviate the adverse responses of radiotherapy and chemotherapy, improve the patients’ life quality, and reduce the cancer recurrence [76].

### Huanglian Jiedu Tang

Huanglian Jiedu Tang (HJT), a classic herbal formula, consists of four herbs: *Coptis* Rhizome (Huang Lian)*, Phellodendri chinensis* Cortex (Huang Bai)*, Scutellariae* Radix (Huang Qin), and *Gardeniae* Fructus (Zhi Zi), with equal proportion. In this prescription, the first three herbs listed above have the roles of purging fire and removing toxin, and function as monarch, minister and assistant in the formula, respectively. The decoction, previously acting as an anti-inflammatory agent, is widely used for treating dermatitis, gastritis, liver injuries, and bleeding of the intestines and uterus [77]. HJT has been extensively used in TCM practice even though their mechanisms of action remain unclear. It was reported that HJT could effectively cause hepatoma cell cycle arrest by upregulating the inactive form of Cdc2 and Cdc25, and downregulating the levels of Bcl-2 and Bcl-xL. Moreover, HJT exerted antitumor effect through increasing the expression of Bax and Bak and decreasing the expression of Bcl-2 and Bcl-xL via inhibition of the NF-κB activity, and consequently inducing the mitochondria-dependent apoptosis in hepatoma cells [78]. HJT could inhibit primary myeloma cell proliferation and survival, and induce the cell apoptosis via a mitochondria-mediated pathway. Further studies revealed that *Scutellaria* Radix and one of its major compounds baicalein were responsible for the anticancer effect of HJT on myeloma [79]. Experiments were conducted on evaluating the preventive effect of oral administration of HJT on stomatitis and diarrhea induced by cytotoxic drugs in patients with acute leukemia. It was found that the incidence of mucositis and diarrhea was apparently lower than the control group [80]. In addition, HJT shows remarkable chemopreventive effect with low toxicity on colon cancer by inhibiting COX-2, which is involved in the production of prostanoids that could promote inflammation and tumorigenesis, but not COX-1, a constitutively expressed enzyme for normal functions of many organs [81], indicating the advantages of HJT over non-steroidal anti-inflammatory drugs, which inhibit both COX-1 and COX-2.

**Table 2:** Anticancer effects and mechanisms of HCD herb-containing formulas

HCD formulas	Components	Cancer types (cell lines)	Cellular effects	Molecular mechanisms	References
Yanshu Injection	*Sophorae flavescentis* Radix*, Smilacis glabrae* Rhizoma	Hepatocellular carcinoma (HepG2), breast cancer (MDA-MB-231), bladder cancer (T24)	Inhibiting proliferation, adhesion, invasion, migration and metastasis inducing apoptosis and autophagy, modulating immune response	↓TGF-β1, ↓TGFBR1	[73–76]
Huanglian Jiedu Tang	*Coptidis rhizome, Phellodendri chinensis* Cortex*, Scutellariae* Radix, and *Gardeniae Fructus*	Hepatocellular carcinoma (HepG2, PLC/PRF/5), myeloma (U266, NOP-2, AMO1, ILKM2)	Inhibiting proliferation, inducing apoptosis and cycle arrest	↑p-Cdc2, ↑p-Cdc25C, ↓Cdc2, ↓Cdc25C, ↓Cyclin A, ↓Cyclin B1, ↑Bak, ↓Bcl-2, ↓Bcl-XL, ↑IKB-α, ↓NF-κB, ↓IL-6, ↓XIAP, ↑caspase-3, ↑caspase-9	[78–81]
Jiedu Xiaozheng Yin	*Hedyotis diffusa Willd, Cremastrae pseudobulbus, Pleiones pseudobulbus, Prunellae* Spica and *Sophorae flavescentis* Radix	Hepatocellular carcinoma (HepG2, PLC/PRF/5, Huh7)	Inhibiting proliferation, inducing apoptosis, cycle arrest at G0/G1 phase and loss of plasma membrane asymmetry, decreasing mitochondrial membrane potential	↑Cyclin D, ↑Cyclin E, ↓C-myc, ↓Cyclin D1, ↓PCNA, ↓Bmi1, ↑p16, ↑caspase-3, ↑caspase-9, ↑Bax/Bcl-2	[19, 82–84]
PHY906	*Scutellariae* Radix, *Glycyrrhizae Radix Et Rhizoma, Jujubae Fructus, Paeoniae Radix Alba*	Hepatocellular carcinoma (HepG2)	Inducing apoptosis	↑FasL, ↑FasR, ↑hMCP1, ↑AMPKα-T172-P, ↑ULK1-S555-P, ↑ERK1/2-P	[86–89]
Feiji Recipe	*Astragali Radix, Glehniae Radix, Ophiopogonis Radix, Asparagi Radix, Poria, Ligustri Lucidi Fructus, Selaginella doederleinii Hieron, Coicis semen, Salivae Chinensis Herba, Epimedii Folium, Trichosanthis Pericarpium, Paris polyphylla Smith var. chinensis (Franch.) Hara, Ranunculus ternatus, Pinelliae Rhizoma, Cremastrae Pseudobulbus, Arisaematis Rhizoma Preparatum, Houttuyniae Herba, and Prunellae Spica*	Lung cancer (LLC)	Inhibiting proliferation, intervening immune escape	↓CD4+CD2+Tr, ↓VEGF, ↓Scd44V6, ↓TGR-β1, ↓IL-10	[102, 103]
YiQi ChuTan Recipe	*Panacis Quinquefolii* Radix*, Ophiopogonis* Radix, *Phellodendri Chinensis* Cortex, *Cremastrae Pseudobulbus, Stephaniae Tetrandrae* Radix*, Pinelliae* Rhizoma, Gynostemma pentaphyllum (Thunb.) Makino, and *Hominis Placenta*	Lung cancer (A549, LLC)	Inhibiting proliferation and metastasis, reversing EMT	↓GRP78, ↓smad2/3, ↓SRC/MAPK, ↑Caspase-4, ↑DNA-PK, ↓Hspd1, ↓PH, ↓PDI, ↓EG433182, ↓HSPA 5 precursor, ↓HSPA 9, ↓PP1, ↓PRDX-1, ↓PRDX-6	[104–106]
Jianpi Yangzheng Xiaozheng Recipe	*Codonopsis* Radix*, Atractylodis Macrocephalae* Rhizoma*, Poria, Dioscoreae* Rhizoma*, Coicis semen, Citri Reticulatae* Pericarpium*, Aucklandiae* Radix*, Angelicae Sinensis* Radix*, Paeoniae* Radix Alba*, Smilacis Chinae Rhizoma, Salivae Chinensis* Herba, and *Glycyrrhizae Radix Rhizoma Praeparata Cum Melle*	Gastric cancer (MGC-803)	Inducing apoptosis and autophagy	↑Bax, ↓Bcl-2, ↓cyclin D1, ↓cyclin D2, ↓cyclin D3, ↑Fas, ↓procaspase-3, ↓procaspase-8, ↓procaspase-9, ↑cleaved-PARP, ↑Beclin-1, ↑LC3 II	[107]
Fuzheng Qingjie Recipe	*Astragali Radix, Ligustri Lucidi Fructus, Ganoderma, Dioscoreae Rhizoma, Prunellae Spica and Hedyotis diffusa Willd*	Hepatocellular carcinoma (HepG2)	Inducing apoptosis	↑caspase-9, ↑caspase-3, ↑P38 MAPK, ↑Bax, ↓Bcl-2	[108]
Baihe Recipe	*Solanum lyratum* Thunb., *Hedyotis diffusa Willd, Agrimoniae* Herba*, Codonopsis* Radix, and *Poria,*	Gastric cancer (BGC-823)	Inhibiting proliferation and metastasis	↓VEGF, ↓p53	[109]
Weikangfu Granule	*Curcumae* Radix*, Astragali* Radix*, Glycyrrhizae Radix Et Rhizoma, and Poria*	Sarcoma (S180)	Inducing apoptosis and cell cycle arrest at G0/G1 phase, modulating immune response	↑p53, ↑Bax, ↓Bcl-2	[110, 111]

### Jiedu Xiaozheng Yin

Jiedu Xiaozheng Yin (JXY), an anticancer decoction of TCM possessing heat-clearing and detoxification properties, consists of *Hedyotis diffusa* (Bai Hua She She Cao)*, Cremastrae pseudobulbus Pleiones pseudobulbus* (Shan Ci Gu)*, Prunellae* Spica (Xia Ku Cao) and *Sophorae flavescentis* Radix (Ku Shen). The formula exerted growth inhibitory effect on HepG2 hepatocarcinoma cells in a dose-dependent manner via increasing the expression of G1-related cyclins D and E [82]. However, the constituents that responsible for the antitumor effects are still largely unknown. In vitro experiments indicated that JXY inhibited the proliferation of gastric carcinoma cell line and promoted apoptosis via mitochondrial pathway in the hepatic carcinoma cancer cells. The ethanol extract of JXY (EE-JXY) decreases the viability of human umbilical vein endothelial cells and the tube formation capacity. Moreover, EE-JXY inhibits angiogenesis in chick chorioallantoic membrane and decreases microvessel density in the xenograft tumor. Further results demonstrated that JXY inhibited angiogenesis by downregulating VEGF-A and VEGFR-2 expression [19]. Recent studies reported that ethyl acetate extraction of JXY significantly inhibited hepatoma cell growth both in vitro and in the mouse xenograft model through arresting cancer cells at G0/G1 phase, inhibiting angiogenesis, and inducing cancer cell apoptosis, which may involve the suppression of the Bmi1 and Wnt/β-catenin signaling pathways [83]. A clinical study was conducted on hepatic carcinoma in III stage patients treated with JXY for 7 days before operation and Fuzheng Yiliu recipe after operation for 2 years. The results demonstrated that administration of compound Chinese herbal medicines in peri-operational period significantly decreased the recurrence rate, improved patients’ immune function and increased the cumulative survival rate [84].

### PHY906

PHY906, derived from a famous TCM formula called Huang Qin Tang, is composed of four herbs: *Scutellariae* Radix (Huang Qin), *Glycyrrhizae* Radix Et Rhizoma (Gan Cao)*, Jujubae Fructus* (Da Zao)*, and Paeoniae Radix Alba* (Shao Yao), with a ratio of 3:2:2:2. Huang Qin Tang has been used for more than 1000 years in TCM in treating various gastrointestinal discomfort, such as abdominal cramps, vomiting, diarrhea and nausea [85]. Although PHY906 alone has little antitumor effect, it was developed to be an effective TCM recipe for the relief of gastrointestinal toxicity and improvement of the antitumor efficacy of chemotherapeutic drugs, which has been proven both in preclinical animal models and in clinical studies. In a phase I clinical study, it was found that PHY906 could increase the therapeutic outcomes of capecitabine by reducing side effects such as diarrhea in patients with advanced pancreatic cancer, colon cancer, cholangiocarcinoma, or esophageal cancer [86]. In a phase II study, combination administration of PHY906 and capecitabine for patients with advanced pancreatic cancer resulted in a well tolerate and response of the treatment and improved indices of quality of life, including fatigue, loss of appetite, nausea, impaired sense of well-being, and diarrhea [87]. Studies revealed that PHY906 possessed a wide range of pharmacological activities due to its multiple components and mechanisms, including inhibitory activities on multi-drug resistant protein (MDR) and CYP450 which could result in enhancement of cellular uptake of chemotherapeutic agents, inhibitory activities on NF-κB and matrix metalloproteases which could inhibit angiogenesis and enhance the antitumor effect of chemotherapeutic agents, and inhibition of tachykinin NK-1, opiate δ receptors and acetylcholine esterase which may contribute to the improvement of quality of life [88]. Although PHY906 does not directly protect the initial impairment of intestine caused by irinotecan, it can effectively ameliorate inflammatory responses through inhibiting multiple inflammation related targets, including TNF-α-induced NF-κB-mediated transcriptional activity and COX-2 and iNOS enzyme activity. In addition, PHY906 remarkably promotes the recovery of damaged intestinal mucosa by increasing the proliferation of progenitor or stem cells and the growth of the crypts through potentiating Wnt/β-catenin signaling activity [89]. This suggests that herbal medicines with multiple components and molecular targets could be promising in future drug discovery and development for the potential management of complicated diseases.

## Conclusions and perspectives

Organisms, at either cellular, organ, or organismal levels, are complex systems featuring redundant networks, self-organization and adaptation to the environment. Similarly, malignant cancers evolve to be a complex system with highly genetic diversity and enormous capability of adaptation to selective pressure [90]. Chinese medicinal herbs and the person-based formulas contain hundreds even thousands of compounds, which may regulate the activities or expression of a broad spectrum of proteins. In this regard, Chinese herbal medicine might be a promising approach for the management of multifactorial chronic diseases including cancers. Since the accumulation of heat and toxins plays a key role in the occurrence and development of cancers according to TCM theory, HCD herbs are commonly prescribed in TCM formulas for the treatment of cancer. Increasing evidences have shown that decoction or components of HCD herbs or HCD herbs-containing formulas exhibited favorable anticancer effects directly or through enhancing the activities of chemotherapeutic drugs. However, huge efforts still need to be deployed in this field to bring the most potentials of HCD herbs for cancer treatment, including (1) further evaluation of anticancer efficacy of HCD herbs and formulas, particularly using xenograft animal tumor models; (2) further identification of major component(s) in HCD herbs responsible for the anticancer activity since many of them still have not been identified; (3) investigation of the underlying molecular mechanisms for the anticancer effects of HCD herbs and formulas, particular using cutting-edge technologies for complex sample analysis, e.g. proteomics and metabolomics approaches, since herbs or formulas may have complex mechanisms of action; (4) studies on the adjuvant anticancer activity of HCD herbs and formulas, e.g. sensitizing cancer cells to chemo- or radiotherapy, reversing multidrug resistance, reducing chemotherapy side effects, etc.; (5) studies on the acute and chronic toxicity of HCD herbs extracts and the purified components are also highly demanded. In summary, HCD herbs, formulas and the purified components have highly potential to be developed as anticancer agents used alone or in combination with other therapeutic methods.

## Abbreviations

AATG4B: autophagy related 4 homolog B
ACTA2: smooth muscle alpha (α)-2 actin
AKAP12: A-kinase anchor protein 12
AMPK: AMP-activated protein kinase
AP-1: activator protein 1
[Ca^2+^]_i_: intracellular calcium
CCNA2: cyclin A2
CDC: cyclin-dependent kinases
COX-2: cyclooxygenase 2
CREB: cAMP response element-binding protein
DNA-PK: DNA-dependent protein kinase
ERK: extracellular signal-regulated kinases
FAK: focal adhesion kinase
FasL: Fas ligand
FOXO: forkhead box O proteins
GlyRS: glycyl-tRNA synthetase
GRP78: glucose-regulated protein 78
GSK3β: glycogen synthase kinase 3β
HCD: heat-clearing and detoxicating
HIF-1α: hypoxia-inducible factor 1α
hMCP1: human monocyte chemoattractant protein-1
hnRNP-1: heterogeneous nuclear ribonucleoprotein 1
HP1: heterochromatin protein 1
HSP 70: heat shock protein 70
hTERT: human telomerase reverse transcriptase
IFN: interferons
IGF-1: insulin-like growth factor 1
IGF-IR: type 1 IGF receptor
IKB: IκB
IKK: IκB kinases
IL-1β: interleukin 1β
JNK: c-Jun N-terminal kinases
KDR: kinase insert domain receptor
LC3: microtubule-associated protein 1A/1B-light chain 3
MAPK: mitogen-activated protein kinases
mFasL: membrane-bound FasL
MMP: matrix metalloproteinases
mTOR: mechanistic target of rapamycin protein
MX1: an interferon-induced GTP-binding protein
NAT: N-acyltransferases
NF-κB: nuclear factor-κB
ODC1: ornithine decarboxylase 1
p70S6K: p70S6 kinase
PARP: poly (ADP-ribose) polymerase
p-Cdc: phosphorylated CDC
PCNA: proliferating cell nuclear antigen
PDEF: prostate-derived Ets transcription factor
PDI: protein disulfide isomerase
PI3K: phosphoinositide 3-kinase
PP1: protein phosphatase 1
PPAR γ: peroxisome proliferator-activated receptor γ
PPase: protein Phosphatase
PRDX: peroxiredoxins
PSA: prostate-specific antigen
Rb: retinoblastoma protein
RKIP: Raf kinase inhibitor protein
sFasL: soluble form of FasL
STAP: stellate cell activation-associated protein
STAT3: signal transducer and activator of transcription 3
TCM: traditional Chinese medicine
TCTP: translationally controlled tumor protein
TGFBR1: transforming growth factor β receptor 1
TIMP: metallopeptidase inhibitors
TNF-α: tumor necrosis factor α
TRAIL: TNF-related apoptosis-inducing ligand
ULK1: Unc-51 like autophagy activating kinase 1
VEGF: vascular endothelial growth factor
WTX: Wilms tumor gene on X chromosome
XIAP: X-linked inhibitor of apoptosis protein
